# Supplementary material for: High-throughput phenotypic screen and transcriptional analysis identify new compounds and targets for macrophage reprogramming
Source: Nat Commun. 2021 Feb 3;12:773. doi: 10.1038/s41467-021-21066-x (PMC7858590; doi:10.1038/s41467-021-21066-x)
Supplement: Supplementary file 1 — Supplementary Information [file 41467_2021_21066_MOESM1_ESM.pdf]

SUPPLEMENTARY INFORMATION

High-throughput phenotypic screen and transcriptional analysis identify new compounds and targets for macrophage reprogramming

Guangan Hu[1*], Yang Su[1], Byong Ha Kang[2], Zhongqi Fan[1], Ting Dong[1], Douglas R. Brown[1], Jaime Cheah[1], Karl Dane Wittrup[2] and Jianzhu Chen[1*]

[1]Koch Institute for Integrative Cancer Research and Department of Biology, Massachusetts Institute of Technology, 77 Massachusetts Avenue, Cambridge, MA 02139, USA
[2]Koch Institute for Integrative Cancer Research and Department of Biological Engineering, Massachusetts Institute of Technology, 77 Massachusetts Avenue, Cambridge, MA 02139, USA

This file includes:
Supplementary Figures 1-13
Supplementary Table 1
Supplementary Table 2
Supplementary Table 3

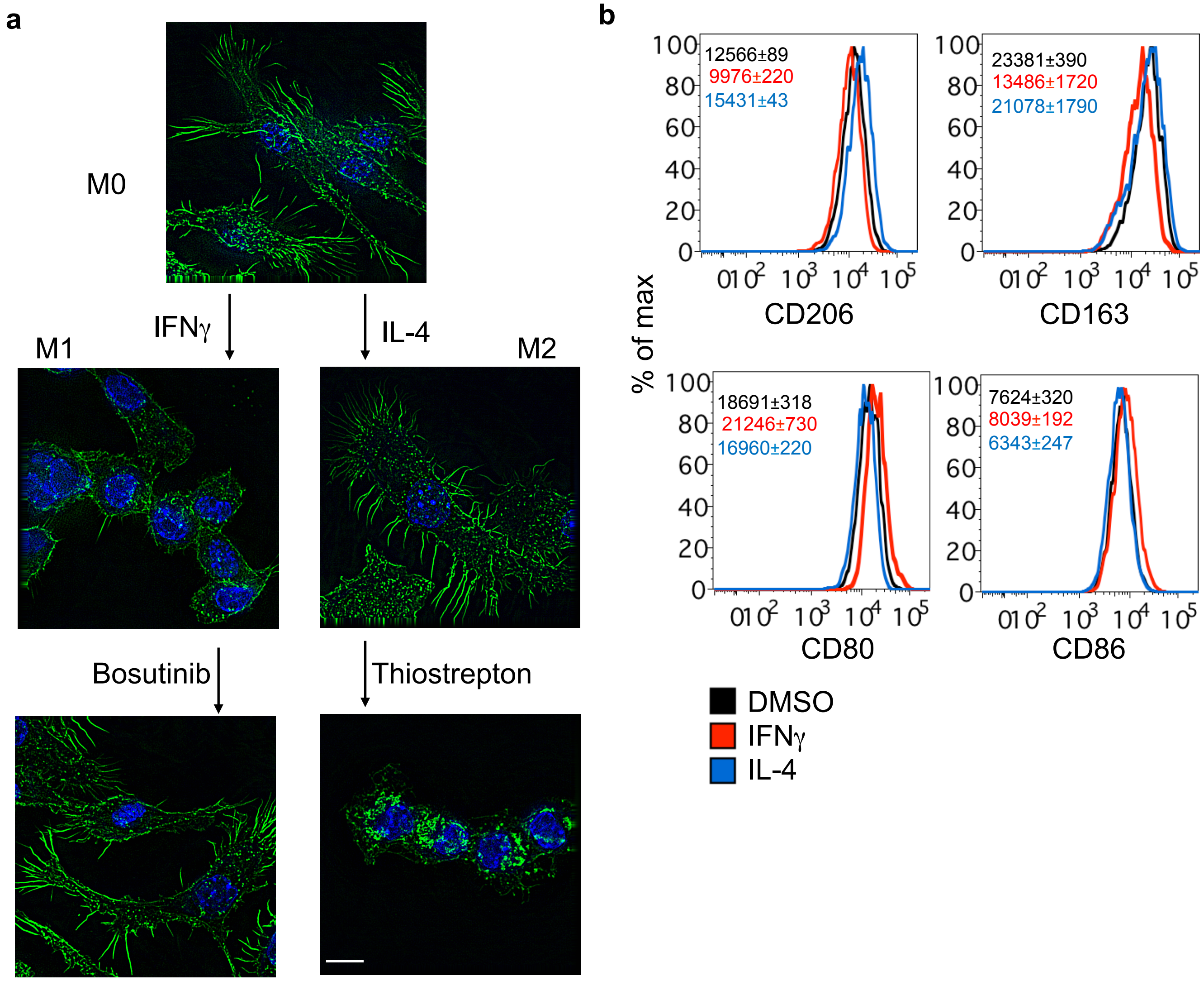

Supplementary Fig. 1. Morphology and phenotypes of activated macrophages. **a**, F-actin staining of M1- and M2-like macrophages. hMDMs were induced to become M0 by M-CSF. The resulting macrophages were polarized to M1 by IFNγ or M2 by IL-4. Then, M1 macrophages were treated with M2-activating compound bosutinib (1 μM) for 24 hrs, and M2 macrophages were treated with M1-activating compound thiostrepton (2.5 μM) for 24 hrs. F-actin was stained by propidium iodide and images were acquired by fluorescent microscopy with 60x objective. Blue is DAPI staining of cell nuclei. Representative data were shown from two independent experiments. Scale bar: 20 μm. **b**, CD163, CD206, CD80 and CD86 expression by hMDM treated with DMSO (black), or IFNγ (red) or IL-4 (blue) quantified by flow cytometry. Shown are the representative staining profiles from three independent experiments. The numbers show mean fluorescent intensity (MFI) ± standard error of the mean (SEM) for n=3 samples per group.

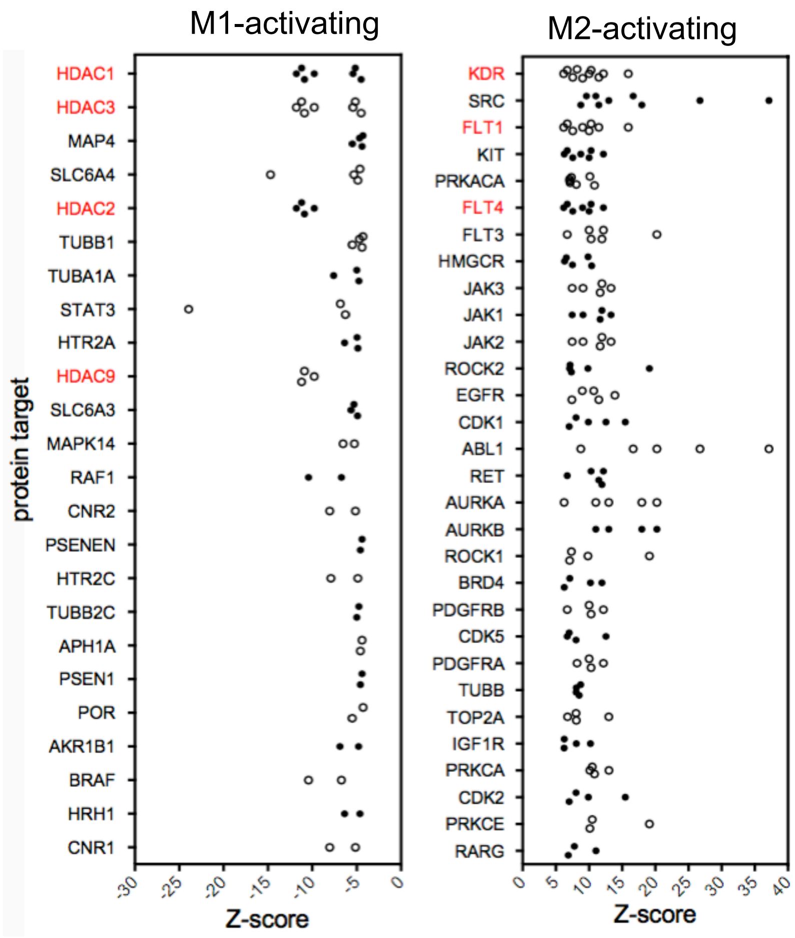

Supplementary Fig. 2. The top list of proteins that are targeted by M1-activating and M2-activating compounds. Histone deacetylases and VEGF receptors are highlighted by color. Each dot represents one compound in the compound library.

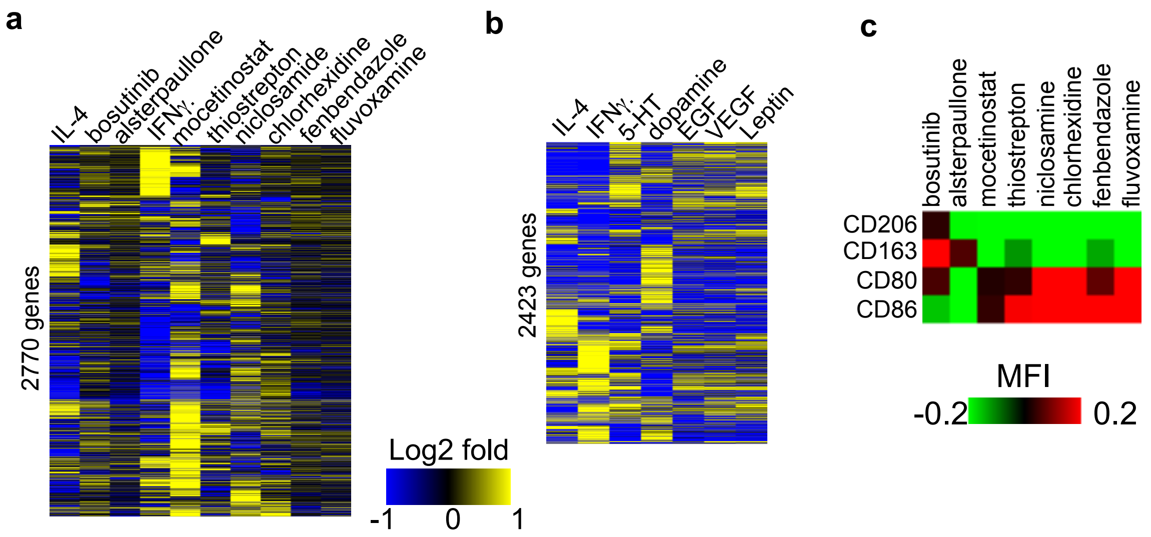

Supplementary Fig. 3. Comparison of the differentially expressed genes induced by selected compounds (**a**), ligands for novel pathways (**b**), and controls (IL-4 and IFNγ). **c**, Changes of the selected M1 markers (CD80 and CD86) and M2 markers (CD206 and CD163) at protein level induced by compound as assayed by flow cytometry. Shown are the changes of the relative mean fluorescence intensity (MFI) to controls. 0.2 refers to 20% increase in MFI.

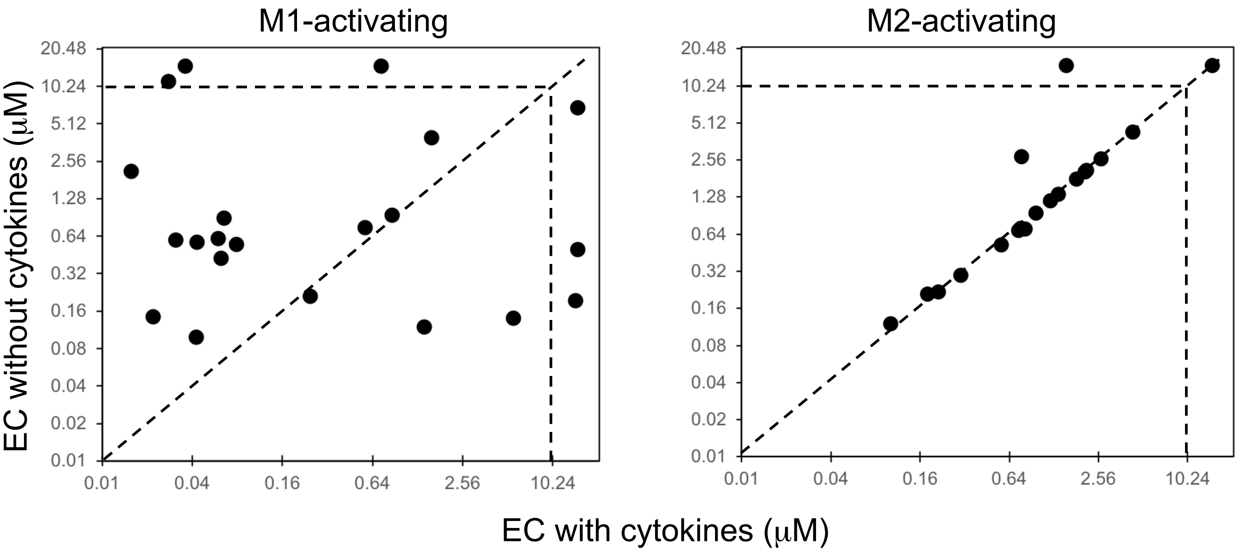

Supplementary Fig. 4. Comparison of EC of 21 M1-activating and 19 M2-activating compounds in the presence or absence of the polarizing cytokines. hMDMs were differentiated into either M2 by IL-4 plus IL-13 for the dosage assays of M1-activating compounds, or M1 by IFNγ plus TNFα for the dosage assays of M2-activating compounds. The effective concentrations were calculated by the Michaelis-Menten equation and plotted. Data were summarized from 2 independent experiments.

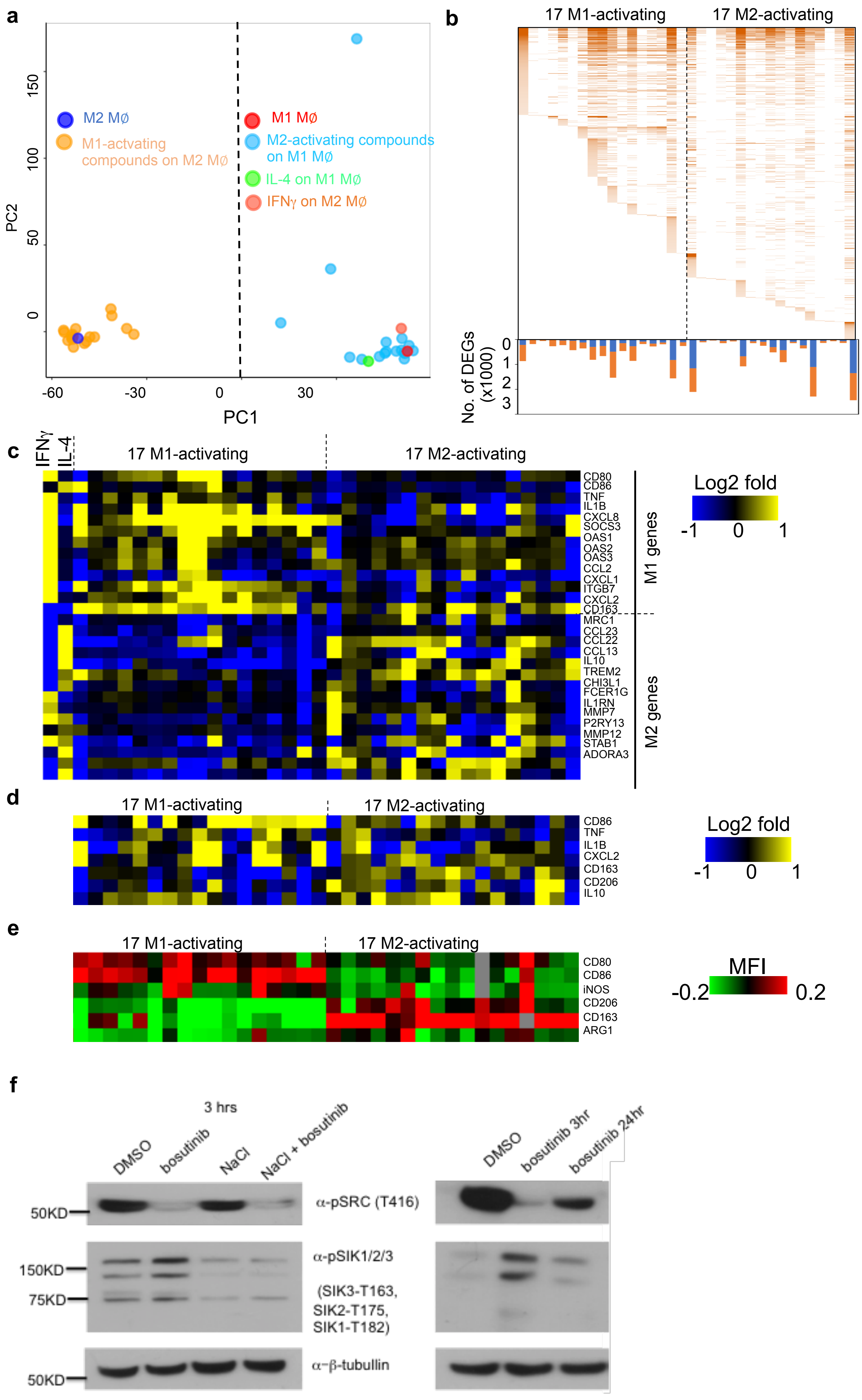
a
M2 Mø
M1-actvating compounds on M2 Mø
M1 Mø
M2-activating compounds on M1 Mø
IL-4 on M1 Mø
IFNγ on M2 Mø
PC2
PC1
b
17 M1-activating
17 M2-activating
No. of DEGs (x1000)
c
IFNγ
IL-4
17 M1-activating
17 M2-activating
CD80
CD86
TNF
IL1B
CXCL8
SOCS3
OAS1
OAS2
OAS3
CCL2
CXCL1
ITGB7
CXCL2
CD163
MRC1
CCL23
CCL22
CCL13
IL10
TREM2
CHI3L1
FCER1G
IL1RN
MMP7
P2RY13
MMP12
STAB1
ADORA3
M1 genes
M2 genes
Log2 fold
d
17 M1-activating
17 M2-activating
CD86
TNF
IL1B
CXCL2
CD163
CD206
IL10
Log2 fold
e
17 M1-activating
17 M2-activating
CD80
CD86
iNOS
CD206
CD163
ARG1
MFI
f
3 hrs
DMSO
bosutinib
NaCl
NaCl + bosutinib
DMSO
bosutinib 3hr
bosutinib 24hr
50KD
150KD
75KD
50KD
α-pSRC (T416)
α-pSIK1/2/3
(SIK3-T163, SIK2-T175, SIK1-T182)
α-β-tubullin

Supplementary Fig. 5. Reprogramming of differentiated macrophages by selected compounds. **a**, Principal component analysis of global transcriptional responses of hMDMs to 17 M1-activating and 17 M2-activating compounds. The samples are the same as those in Figure 4a. **b**, Functional enrichment analysis of DEGs induced by each compound. Shown is the assembled heatmap and number of up-regulated (orange) and down-regulated (blue) DEGs (bottom panel). **c**, Comparison of relative transcript levels of the selected M1 and M2 genes following compound treatment based on RNA-seq. **d**, Comparison of the transcript levels of the selected M1 and M2 genes following compound treatment as measured by quantitative PCR. **e**, Comparison of the protein levels of the selected M1 and M2 markers following compound treatment as measured by flow cytometry. Shown are the relative MFI change to controls. 0.2 refers to 20% increase in MFI. The order of M1-activating and M2-activating compounds in b-e is the same as in Figure 4a. **f**, bosutinib regulates the phosphorylation of SRC and salt-induced kinases. THP1 cells were treated with DMSO (control), bosutinib (1 μM) for 3hrs, or NaCl (additional 200 mM) or bosutinib plus NaCl for 3 (left panel) or 24 hrs (right panel). Cell lysates were subjected to Western blotting with the indicated antibodies.

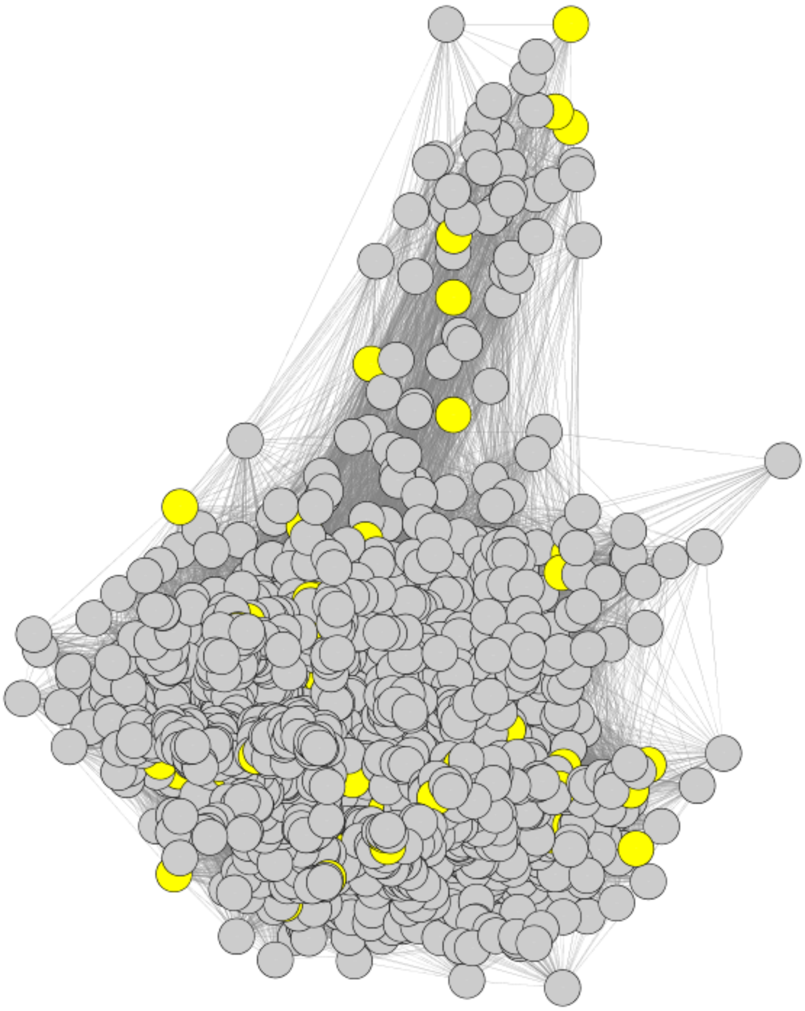

Hub genes

Gene	Degree
GBP1	689
KIAA1244	659
FAM26F	617
COLEC12	596
GBP4	582
MYO1G	554
TYMP	546
GCH1	528
SNX10	514
CNIH4	508
STAT1	498
MCOLN1	491
NISCH	486

Hub TF

Gene	Degree
STAT1	498
MXD4	456
TSC22D1	427
ZNF814	413
BNC2	413
ZNF331	383
IRF1	373
ZNF440	347
HES2	345
ZBTB8A	325
BATF2	319
MAX	319
NFIX	305

Supplementary Fig. 6. Macrophage activation network. The network was inferred by ARACNe (Margolin et al. 2006). The top 10% central hub gene network was visualized by Cytoscape (Shannon et al. 2003). Yellow marked nodes are transcription factors (regulators). Top 10 central hubs and top 10 central TF hubs are listed.

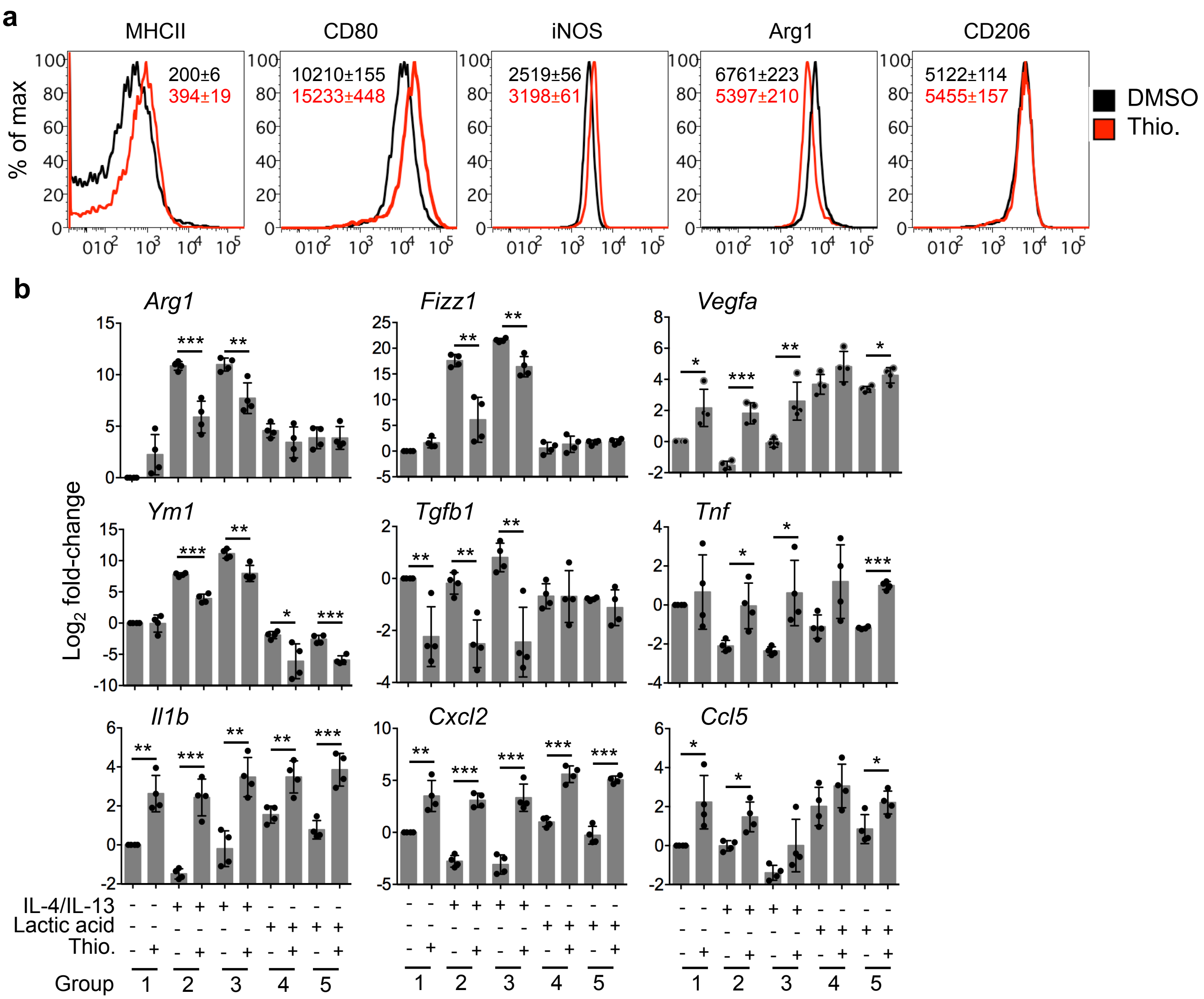

Supplementary Fig. 7. Thiostrepton inhibits the development and function of M2-like macrophages *in vitro.* **a**, Mouse BMMs were cultured with B16F10 tumor cell conditioned medium (CM) for 24 hrs first and then treated with 2.5 μM thiostrepton for another 24 hrs (group 3 from Fig. 5d). Expression of MHCII, CD80, iNOS, Arg1 and CD206 were quantified by flow cytometry. Shown are representative staining profiles of treated (red) and untreated (black) TAMs from two independent experiments. **b***,* Mouse BMMs were not treated or treated with 2.5 μM thiostrepton for 24 hrs in normal medium (group 1), or polarized with IL-4/IL-13 in the absence or presence of 2.5 μM thiostrepton for 24 hrs (group 2), or polarized with lactic acid in the absence or presence of 2.5 μM thiostrepton for 24 hrs (group 4). Alternatively, mouse BMMs were polarized with IL-4/IL-13 (group 3) or lactic acid (group 5) for 24 hrs first and then either not treated or treated with 2.5 μM thiostrepton for another 24 hrs. The transcript levels of the indicated genes were quantified by qPCR. Data are presented as mean ± sd from 4 independent samples per group in two independent experiments. * P<0.05, ** P<0.01 and *** P <0.001 by two-sided T-test between untreated and treated groups.

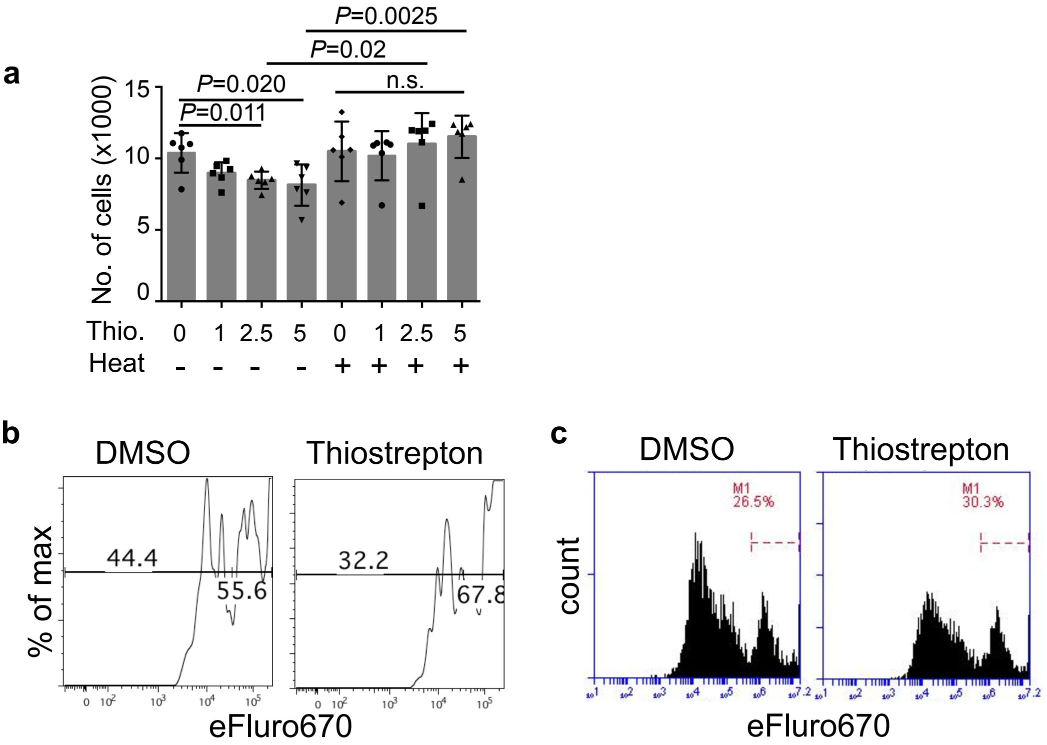

Supplementary Fig. 8. Thiostrepton activates macrophages in vitro. **a**, Mouse BMMs were treated with thiostrepton for 24 hrs (same as Fig. 5e). Conditioned medium (CM) was collected and filtered. B16F10 melanoma cells were cultured for 12 hrs with CM or CM that was heat-inactivated at 95°C for 5 min. The number of tumor cells were quantified by flow cytometry. Data are presented as mean ± sd from two independent experiments. *P*-values are indicated by two-sided T-test. n.s.: not significant. **b-c**, Thiostreption enhances ADCP of macrophages. Mouse BMMs (**b**) or hMDM (**c**) were treated with 2.5 μM thiostrepton for 24 hrs, then co-cultured with equal number of eFluro670 and anti-CD20 labelled human B-cell lymphoma cells for 2 hrs, and analyzed by flow cytometry. Macrophages that have phagocytosed tumor cells are identified as efluro670$^+$ and CD14$^+$. Shown are representative eFluro670 histograms gating on CD14$^+$ macrophages from three different experiments.

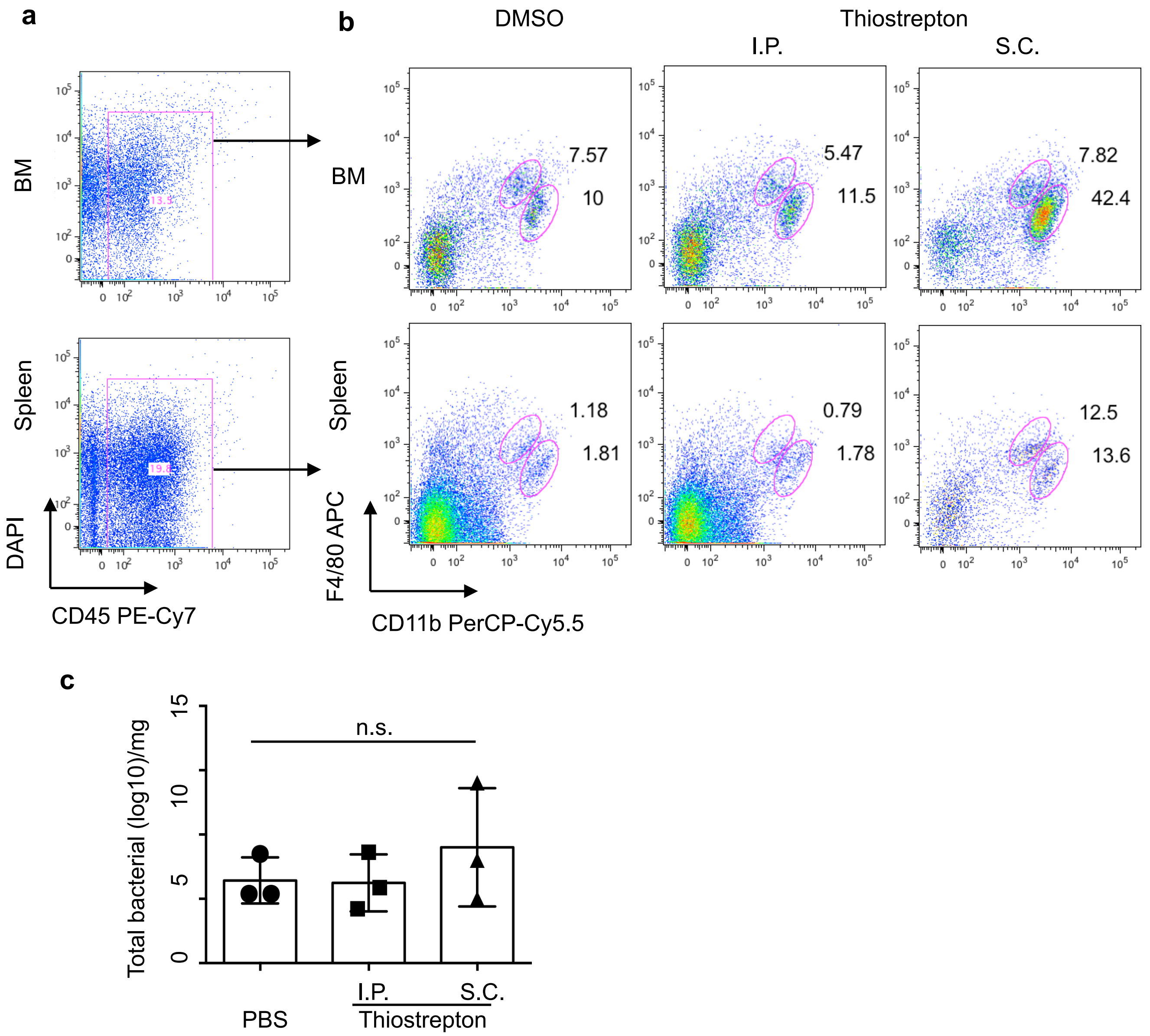

Supplementary Fig. 9. Thiostrepton activates macrophages *in vivo* without altering the total number of gut bacterial counts. **a**, Gating strategy of CD45+ live immune cells in bone marrow (BM) and spleen. **b**, Flow cytometry analysis of macrophages ($F4/80^{+}CD11b^{+}$) and monocytes ($F4/80^{-}CD11b^{+}$) in the bone marrow and spleen of 8-10 weeks old normal B6 mice 6 days post treatment with either DMSO or thiostrepton by I.P. or S.C. (n=3). Shown are representative F4/80 versus CD11b staining profiles gating on $CD45^{+}$ cells from one mouse per group. I.P.: intraperitoneal injection; S.C.: paratumor subcutaneous injection. **b**, Total bacterial counts in the stool sample of mice (n=3). Data shown are mean ± sd. n.s., not significant by two-sided T-test.

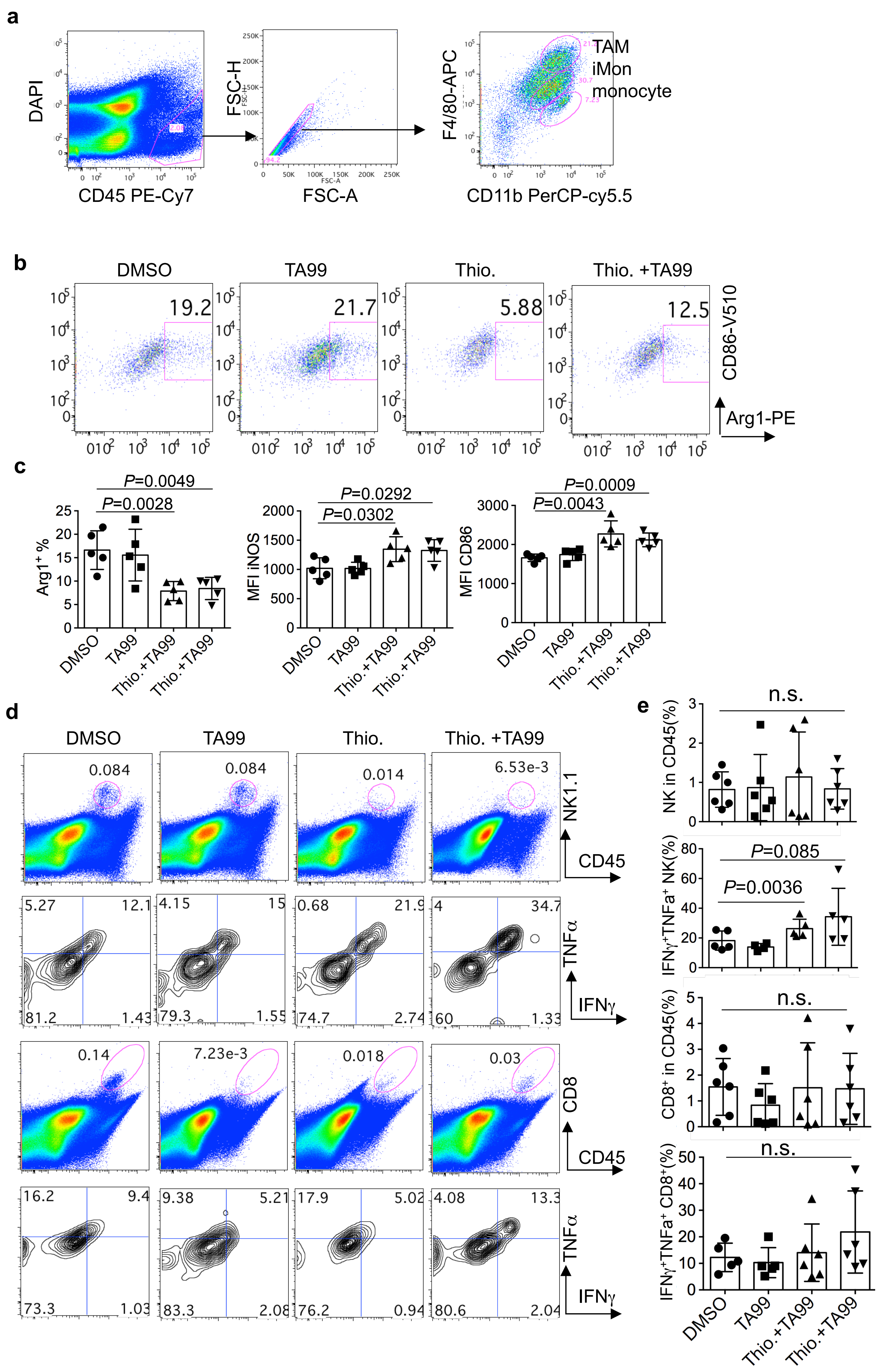
a
DAPI
CD45 PE-Cy7
FSC-H
FSC-A
F4/80-APC
CD11b PerCP-cy5.5
TAM
iMon
monocyte
b
DMSO
TA99
Thio.
Thio. +TA99
19.2
21.7
5.88
12.5
CD86-V510
Arg1-PE
c
P=0.0049
P=0.0028
Arg1+ %
P=0.0292
P=0.0302
MFI iNOS
P=0.0009
P=0.0043
MFI CD86
DMSO
TA99
Thio.+TA99
Thio.+TA99
d
DMSO
TA99
Thio.
Thio. +TA99
0.084
0.084
0.014
6.53e-3
NK1.1
CD45
TNFα
IFNγ
0.14
7.23e-3
0.018
0.03
CD8
CD45
TNFα
IFNγ
e
n.s.
NK in CD45(%)
P=0.085
P=0.0036
IFNγ+TNFa+ NK(%)
n.s.
CD8+ in CD45(%)
n.s.
IFNγ+TNFa+ CD8+(%)
DMSO
TA99
Thio.+TA99
Thio.+TA99

Supplementary Fig. 10. Effect of thiostrepton on macrophages, NK cells and $CD8^+$ T cells *in vivo*. B6 mice bearing subcutaneous B16F10 tumor were treated as in Figure 6. Single cell suspensions were prepared from tumors 18 day after engraftment, stained and analyzed by flow cytometry. **a,** gating strategy for flow analysis for TAM, inflammatory monocyte (iMon) and monocyte for Fig. 6**c**. **b-c** Representative intracellular staining profiles of Arg1 vs. CD86 gated on $F4/80^+CD11b^+$ TAMs (**b**) and summarized data (mean ± sd) from n=5 mice per group from two independent experiments in Fig. 6**a** (**b**). **c** Representative intracellular staining profiles of IFNγ vs. TNFα gated on $CD45^+NK1.1^+$ NK cells (top two rows) and $CD45^+CD8\alpha^+$ T cells (bottom two rows). Samples for T-cell staining were stimulated in vitro by T-cell stimulation cocktail for 4 hrs. **d**, summarized data from n=4-6 mice per group from two independent experiments in Fig. 6**a**. Data shown are mean ± sd. *P* values are based on two-sided T–test and indicated. n.s., not significant.

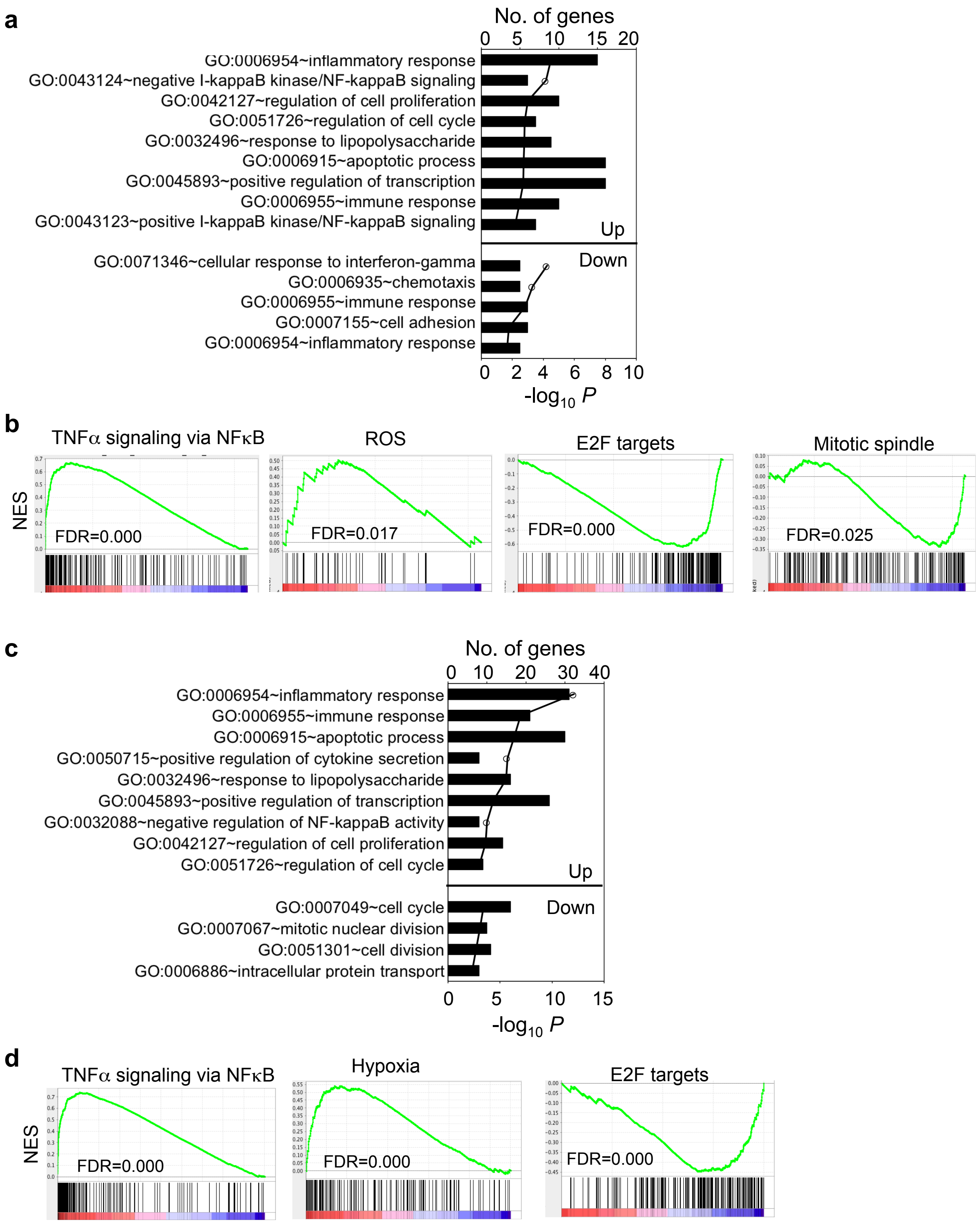

Supplementary Fig. 11. Transcriptional responses of TAMs to thiostrepton *in vivo*. **a**, GO enrichment analysis showing enrichment of certain pathways in the up-regulated and down-regulated genes in TAMs following I.P. administration of thiostrepton or DMSO. GO sets of biological process, number of genes and P-value are shown. Tumor infiltrated macrophages were sorted from tumor issues based on $CD45^{+}F4/80^{+}CD11b^{+}Gr1^{-}$ 18 days after tumor engraftment. Gene expression levels were measured by RNA-seq. **b**, GSEA showing enriched gene sets in TAMs induced by thiostrepton *in vivo* by I.P. administration (FDR q-value <0.05). **c**, GO enrichment analysis showing enrichment of certain pathways in the up-regulated and down-regulated genes in TAMs induced by S.C. administration of thiostrepton or DMSO. GO sets of biological process, number of genes and P-value are shown. **d**, GSEA showing enriched gene sets in TAMs induced by thiostrepton *in vivo* by S.C. administration (FDR q-value <0.05). I.P.: intraperitoneal injection; S.C.: paratumor subcutaneous injection.

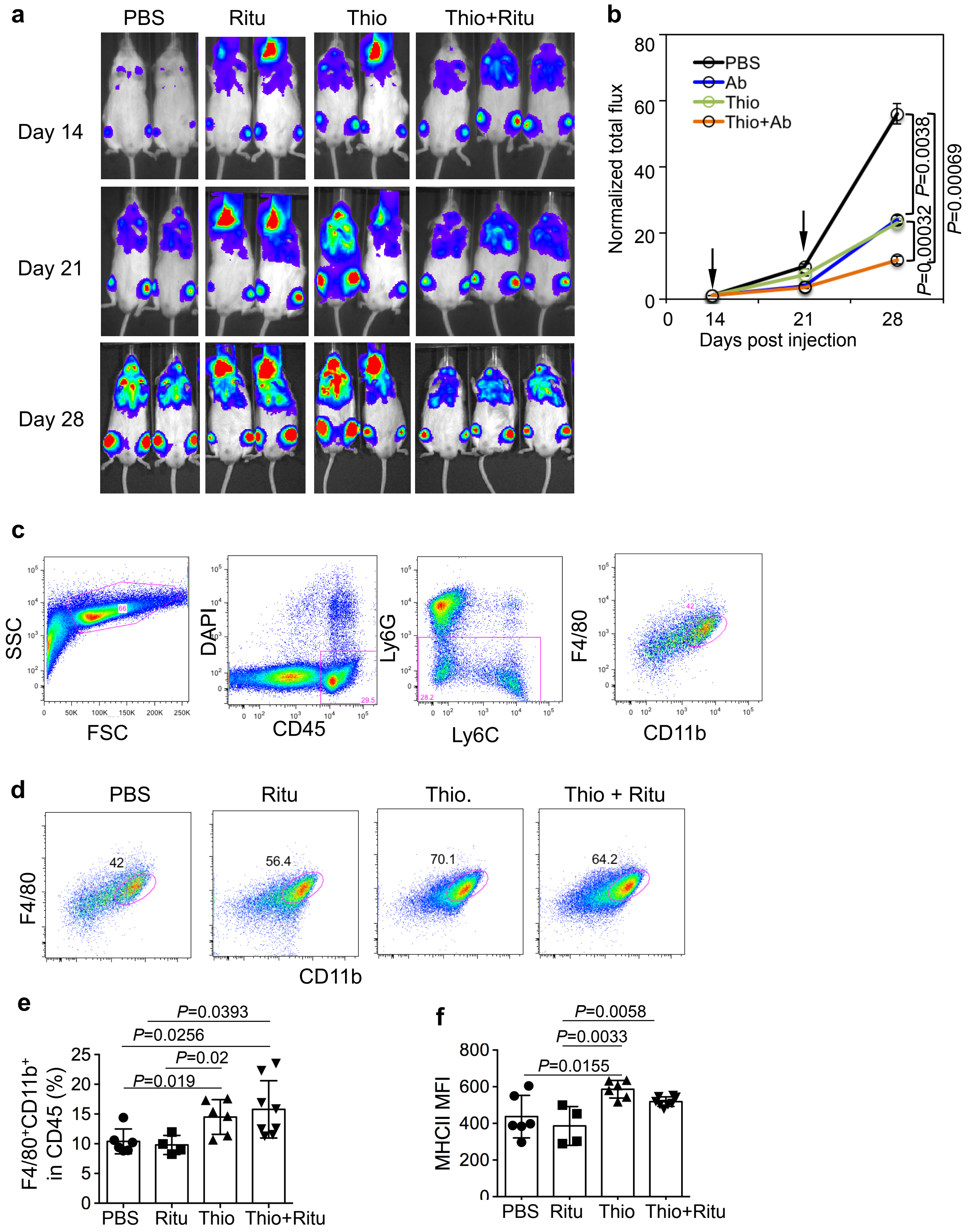

Supplementary Fig. 12. Thiostrepton inhibits tumor growth in the bone marrow. NSG mice were grafted with $1x10^7$ GMB-luc cells and dosed twice at 14 and 21 days later with 0.5 mg/kg Rituximab (Ritu) and/or 300 mg/kg thiostrepton (Thio). Tumor burden was monitored (**a**) and quantified (**b**) by bioluminescence imaging (n=5-6 mice per group). Data are presented as mean ± sem. *P* values are indicated by two-sided T-test. At day 28 post tumor engraftment, bone marrow cells were analyzed by flow cytometry (**c-f**). Shown are representative gating strategy (**c**) and F4/80 versus CD11b staining profiles gating on $CD45^+$ cells (**d**) and and summarized data (mean ± sd) (**e**) from n=4-6 mice per group in two independent experiments. **f**, MHCII expression gating on macrophages**.** Data are presented as mean ± sd from all mice from **e**. *P* values are indicated by two-sided T-test.

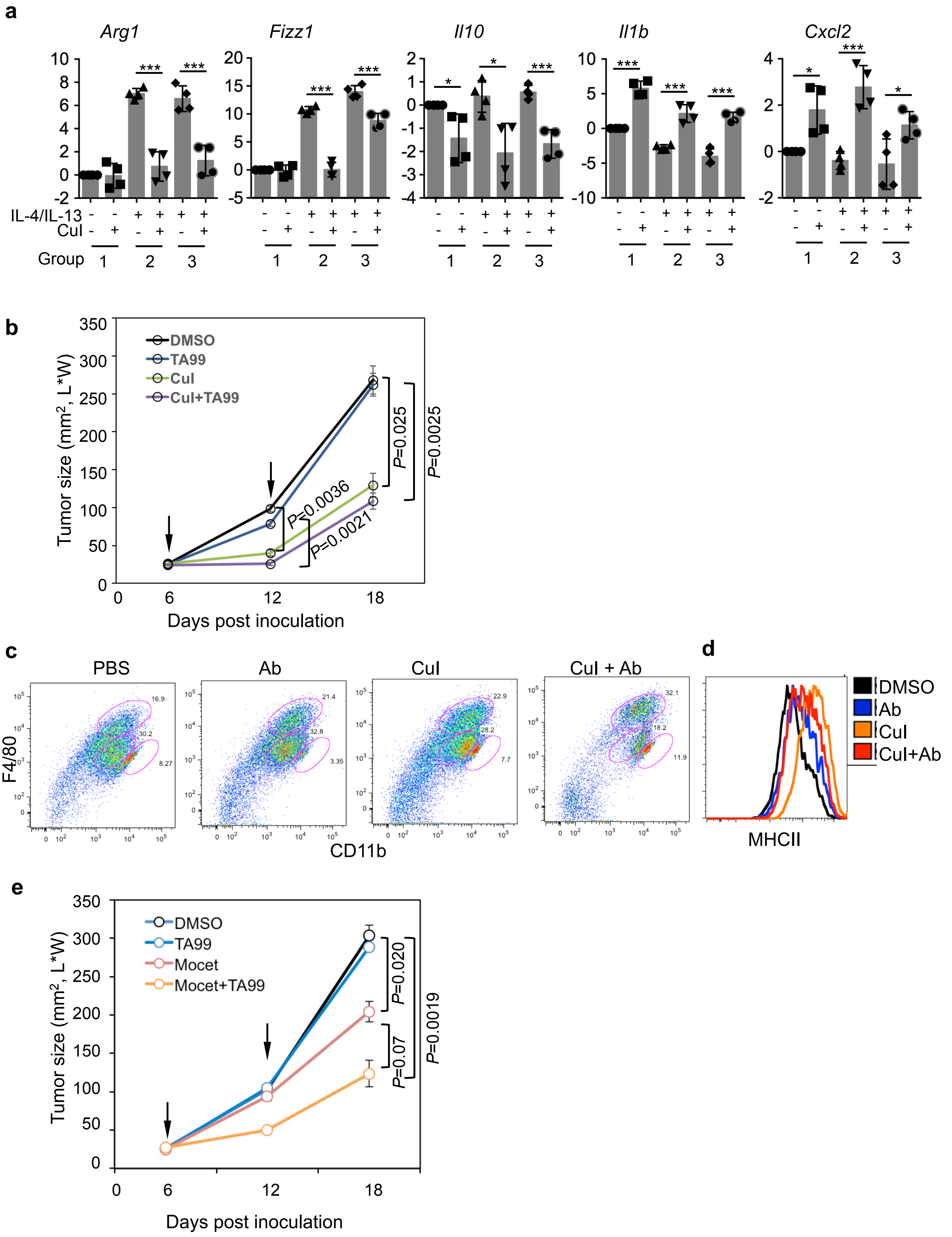
a
Arg1
Fizz1
Il10
Il1b
Cxcl2
IL-4/IL-13
CuI
Group
b
DMSO
TA99
CuI
CuI+TA99
Tumor size (mm², L*W)
Days post inoculation
P=0.025
P=0.0025
P=0.0036
P=0.0021
c
PBS
Ab
CuI
CuI + Ab
F4/80
CD11b
d
DMSO
Ab
CuI
CuI+Ab
MHCII
e
DMSO
TA99
Mocet
Mocet+TA99
Tumor size (mm², L*W)
Days post inoculation
P=0.020
P=0.07
P=0.0019

Supplementary Fig. 13. M1-activating compound, cucurbitacin I, also activates macrophages and inhibits tumor growth. **a**, Cucurbitacin I (CuI) inhibits the development and function of tumor-associated macrophages *in vitro* induced by IL-4/IL-13. Mouse BMMs were not treated or treated with 2.5 μM cucurbitacin I for 24 hours in normal medium (group 1) or in the presence of IL-4/IL-13 (group 2), or mouse BMMs were polarized with IL-4/IL-13 for 24 hours and then either not treated or treated with 2.5 μM CuI for 24 hours (group 3). RNA was isolated and the transcript levels of the indicated genes were quantified by PCR. Data are presented as mean ± sd from 4 independent samples per group in two independent experiments. * P<0.05, ** P<0.01 and *** P <0.001 by two-sided T-test between untreated and treated group. **b**, B16F10 tumor growth in B6 mice treated I.P. with DMSO, TA99, CuI (1 mg/kg) and CuI plus TA99 (n=7-9 mice per group). Data are shown as mean ± sem. P values are based on two-sided T-test and indicated. Arrows show the dosing time. **c-d**, Flow cytometry analysis of TAM ($F4/80^+CD11b^+Ly6C^-Ly6G^-$), inflammatory monocytes ($F4/80^{int}CD11b^+Ly6C^+Ly6G^-$) and monocytes ($F4/80^-CD11b^+Ly6C^+Ly6G^+$) in the tumors of mice treated with DMSO, TA99, CuI, and CuI plus TA99 18 days after tumor engraftment. Shown are representative F4/80 versus CD11b staining profiles gating on $CD45^+$ cells and MHCII histograms gating on macrophages from **c**. Gating strategy is the same as shown in Supplementary Fig. 10**a**. **e**, M1-activating compound, mocetinostat inhibits tumor growth. B16F10 tumor growth in B6 mice treated I.P. with DMSO, TA99, mocetinostat (Mocet)(1 mg/kg) and Mocet plus TA99 (n=5 mice per group). Data are shown as mean ± sem. P values are based on two-sided T-test and indicated. Arrows show the dosing time.

Supplementary Table 1

Dosage information of selected compounds on M0 macrophages

Compound	EC	R square	Max absolu	Category
Taxol	0.10459189	0.416	-6.68139	M1
Cucurbitaci	0.34174063	0.6048	-4.808	M1
Chlorhexidi	1.62736296	0.7308	-13.45	M1
Fenbendaz	0.4703644	0.8408	-12.37	M1
Thiostreptor	3.93691593	0.542	-12.75	M1
Diphenylen	0.67677365	0.4593	-8.509	M1
LE135	0.41887689	0.5289	-8.912	M1
Fluvoxamin	10.6420447	0.8318	-7.38	M1
Niclosamide	0.79658547	0.8077	-8.929	M1
MS275	1.04687704	0.5386	-7.993	M1
Mocetinosta	0.45359214	0.7105	-15.14	M1
Pimozide	1.17941118	0.2042	-8.006	M1
NP-010176	4.03386043	0.5479	-8.156	M1
HMN214	0.10382862	0.6514	-12.51	M1
Celastrol	0.79618903	0.4416	-4.485	M1
Cantharidin	0.17983226	0.2334	-31.36	M1
NVP 231	2.24851869	0.8661	-22.37	M1
FTY720	2.94456442	0.8244	-11.58	M1
Evodiamine	0.3969275	0.9413	-26.36	M1
Penfluridol	2.52355815	0.8439	-4.873	M1
Bostunib	0.09135798	0.3168	23.5	M2
Su11274	0.72193028	0.9476	17.8	M2
Alsterpaullo	0.3076402	0.4391	13.9	M2
ALRESTAT	3.66821661	0.8352	15.05	M2

Supplementary Table 2

Dosage information of selected compounds on differeniated macrophages

Compounds	EC	R-square	Category
Bisantrene dihydroc	1.984795322	0.737	M2
triptolide	0.061894009	0.2428	M2
lovastatin	0.442115573	0.3319	M2
QS 11	0.261082221	0.7666	M2
Regorafenib	2.882594235	0.7596	M2
Sorafenib	0.794071491	0.7332	M2
MLN2238	3.461032864	0.3362	M2
GW-843682X	0.897347267	0.5783	M2
KW 2449	2.16025641	0.8979	M2
Axitinib	0.591495199	0.9957	M2
JTE 013	0.938113208	0.6378	M2
Purmorphamine	2.345142857	0.8143	M2
Arcyriaflavin A	1.342190889	0.6374	M2
Dasatinib	0.761950413	0.6968	M2
NVP-LDE225	2.76599809	0.6752	M2
1-Naphthyl PP1	2.072231834	0.8623	M2
SELAMECTIN	15	0	M2
MGCD-265	0.911173577	0.8933	M2
Bosutinib	0.164371173	0.523	M2
Cantharidin	0.158610234	0.9764	M1
Cucurbitacin I	1.759105431	0.53	M1
Alprostadil	0.056193353	0.1288	M1
HMN-214	1.482432432	0.3942	M1
WP1130	15	0.05	M1
MS275	0.81097561	0.181	M1
SMER 3	0.093980962	0.4302	M1
SCH 79797 dihydro	0.219379028	0.7105	M1
NVP 231	5.744680851	0.4822	M1
Prulifloxacin	15	0	M1
FTY720	0.131265421	0.262	M1
DIHYDROCELASTF	15	0.03	M1
Diphenyleneiodoniu	0.336300175	0.2451	M1
Penfluridol	0.169426434	0.7956	M1
thiostrepton	0.407871889	0.3882	M1
Evodiamine	0.679884726	0.949	M1
MITOXANTRONE H	0.559348161	0.5764	M1
Quinolinium	8.365292011	0.1839	M1
Fenbendazole	0.649293564	0.7447	M1
Niclosamide	1.12595217	0.3178	M1
Taxol	0.128848	0.1359	M1

Supplementary Table 3. qPCR primers

mouse Primer	Sequence
Arg1-F	CATTGGCTTGCGAGACGTAGAC
Arg1-R	GCTGAAGGTCTCTTCCATCACC
Fizz1-F	CAAGGAACTTCTTGCCAATCCAG
Fizz1-R	CCAAGATCCACAGGCAAAGCCA
Vegfa-F	CTGCTGTAACGATGAAGCCCTG
Vegfa-R	GCTGTAGGAAGCTCATCTCTCC
Ym1-F	TACTCACTTCCACAGGAGCAGG
Ym1-R	CTCCAGTGTAGCCATCCTTAGG
Tgfb-F	TGATACGCCTGAGTGGCTGTCT
Tgfb-R	CACAAGAGCAGTGAGCGCTGAA
Tnf-F	GGTGCCTATGTCTCAGCCTCTT
Tnf-R	GCCATAGAACTGATGAGAGGGAG
Il1b-F	ACGGCTGAGTTTCAGTGAGACC
Il1b-R	CACTCTGGTAGGTGTAAGGTGC
Ccl2-F	GCTACAAGAGGATCACCAGCAG
Ccl2-R	GTCTGGACCCATTCCTTCTTGG
Ccl5-F	CCTGCTGCTTTGCCTACCTCTC
Ccl5-R	ACACACTTGGCGGTTCCTTCGA
Cxcl2-F	CATCCAGAGCTTGAGTGTGACG
Cxcl2-R	GGCTTCAGGGTCAAGGCAAACT
Gapdh-F	AGTATGACTCCACTCACGGC
Gapdh-R	GTTCACACCCATCACAAACA
Nos2_F	GAGACAGGGAAGTCTGAAGCAC
Nos2_w	CCAGCAGTAGTTGCTCCTCTTC

human Primer	Sequence
GAPDH-F	GTCTCCTCTGACTTCAACAGCG
GAPDH-R	ACCACCCTGTTGCTGTAGCCAA
TNF-F	CTCTTCTGCCTGCTGCACTTTG
TNF-R	ATGGGCTACAGGCTTGTCACTC
IL1B-F	CCACAGACCTTCCAGGAGAATG
IL1B-R	GTGCAGTTCAGTGATCGTACAGG
CXCL2-F	GGCAGAAAGCTTGTCTCAACCC
CXCL2-R	CTCCTTCAGGAACAGCCACCAA
IL10-F	TCTCCGAGATGCCTTCAGCAGA
IL10-R	TCAGACAAGGCTTGGCAACCCA
CD86-F	TCATTCCCTGATGTTACGAGC
CD86-R	TCTTCCCTCTCCATTGTGTTG
CD163-F	GTGTGATGACTCTTGGGACTTG
CD163-R	AGGATGACTGACGGGATGAG
CD206-F	GACTGATAAGTGGAGGGTGAGG
CD206-R	CCAGAGAGGAACCCATTCG
