# Supplementary material for: High-throughput phenotypic screen and transcriptional analysis identify new compounds and targets for macrophage reprogramming
Source: Nat Commun. 2021 Feb 3;12:773. doi: 10.1038/s41467-021-21066-x (PMC7858590; doi:10.1038/s41467-021-21066-x)
Supplement: Supplementary file 3 — Reporting Summary [file 41467_2021_21066_MOESM3_ESM.pdf]

nature research

Corresponding author(s): Guangan Hu

Last updated by author(s): Jan 5, 2021

Reporting Summary

Nature Research wishes to improve the reproducibility of the work that we publish. This form provides structure for consistency and transparency in reporting. For further information on Nature Research policies, see Authors & Referees and the Editorial Policy Checklist .

Statistics

For all statistical analyses, confirm that the following items are present in the figure legend, table legend, main text, or Methods section.

n/a	Confirmed	
☐	☒	The exact sample size (n) for each experimental group/condition, given as a discrete number and unit of measurement
☐	☒	A statement on whether measurements were taken from distinct samples or whether the same sample was measured repeatedly
☐	☒	The statistical test(s) used AND whether they are one- or two-sided<br>*Only common tests should be described solely by name; describe more complex techniques in the Methods section.*
☒	☐	A description of all covariates tested
☒	☐	A description of any assumptions or corrections, such as tests of normality and adjustment for multiple comparisons
☐	☒	A full description of the statistical parameters including central tendency (e.g. means) or other basic estimates (e.g. regression coefficient) AND variation (e.g. standard deviation) or associated estimates of uncertainty (e.g. confidence intervals)
☐	☒	For null hypothesis testing, the test statistic (e.g. F, t, r) with confidence intervals, effect sizes, degrees of freedom and P value noted<br>*Give P values as exact values whenever suitable.*
☒	☐	For Bayesian analysis, information on the choice of priors and Markov chain Monte Carlo settings
☒	☐	For hierarchical and complex designs, identification of the appropriate level for tests and full reporting of outcomes
☒	☐	Estimates of effect sizes (e.g. Cohen's d, Pearson's r), indicating how they were calculated

Our web collection on statistics for biologists contains articles on many of the points above.

Software and code

Policy information about availability of computer code

Data collection	Detailed in Methods section
Data analysis	bowtie2 2.2.3, RSEM 1.2.15, GSEA 4.0.1, MeV 4.8.1, ARACNe, Cytoscape 3.7.2, BiNGO 3.0.3, Aperio ImageScope 12.3.3, Living Image Software (IVIS Spectrum) 4.7.0, CellProfiler 2, FlowJo 8.8.7, R version (3.5.3) with pakages of RDAVIDWebService v1.28.0 and edgeR 3.24.3.

For manuscripts utilizing custom algorithms or software that are central to the research but not yet described in published literature, software must be made available to editors/reviewers. We strongly encourage code deposition in a community repository (e.g. GitHub). See the Nature Research guidelines for submitting code & software for further information.

Data

Policy information about availability of data

All manuscripts must include a data availability statement. This statement should provide the following information, where applicable:

- Accession codes, unique identifiers, or web links for publicly available datasets
- A list of figures that have associated raw data
- A description of any restrictions on data availability

Raw RNAseq are deposited in the database of Gene Expression Omnibus (GEO) with accession ID: GSE144992 and GSE155551. Compound screening data and differentially expressed genes are provided as Supplementary Data files. All other data that support the findings of this study are available from Source Data file or the corresponding authors upon reasonable request. Certain databases of IUPHAR and Drugbank used for text-mining the information of compounds are accessible via the link guidetopharmacology.org and drugbank.ca. Source data are provided with this paper as a Source Data file.

Field-specific reporting

Please select the one below that is the best fit for your research. If you are not sure, read the appropriate sections before making your selection.

☒ Life sciences ☐ Behavioural & social sciences ☐ Ecological, evolutionary & environmental sciences

For a reference copy of the document with all sections, see nature.com/documents/nr-reporting-summary-flat.pdf

Life sciences study design

All studies must disclose on these points even when the disclosure is negative.

Sample size	Sample sizes were determined according to previous data published by us (Ref.33 and Hu et al. 2019, 45:563 eBiomMedicine) and others using similar biological samples and techniques to detect the significant difference (Chen et al. 2018, 9:873, Nature Communications). All tests were performed with n>=2 independent experiments. In vivo experiments included data from n=4-12 mice for each group and presented as mean with deviation in the graphs and figure legends. Sample sizes and statistical data are reported in figure legends.
Data exclusions	No data were excluded.
Replication	Data reproducibility was confirmed by independent experiments as indicated in the figure legends or methods. Due to the nature of screening, observation in the initial screens (Fig.1) were not replicated, subject to resources. However, we used dosage assay to validate the observations with high accuracy. In addition, observations in the reverse screen (Fig. 3) were performed in two independent experients and in different concentrations and conditions as well, demonstrating similar results.
Randomization	All animals were randomly assigned to different groups for treatments and control as well. Cell culture experiments with cell lines were handled the same way. Bone marrow derived macrophages from one single mouse was taken as one independent sample.
Blinding	The investigators were not blinded to all conditions as they were responsible for both experimental design and data collection. The melanoma tumor sizes were measured by one technician without knowing the information of mouse group.

Reporting for specific materials, systems and methods

We require information from authors about some types of materials, experimental systems and methods used in many studies. Here, indicate whether each material, system or method listed is relevant to your study. If you are not sure if a list item applies to your research, read the appropriate section before selecting a response.

Materials & experimental systems

n/a	Involved in the study	
☐	☒	Antibodies
☐	☒	Eukaryotic cell lines
☒	☐	Palaeontology
☐	☒	Animals and other organisms
☐	☒	Human research participants
☒	☐	Clinical data

Methods

n/a	Involved in the study	
☒	☐	ChIP-seq
☐	☒	Flow cytometry
☒	☐	MRI-based neuroimaging

Antibodies

Antibodies used	1. mouse antibodies: PerCP-Cy5.5-CD11b, clone M1/70; purified and APC-F4/80, clone BM8; APC-Cy7-MHC-II, clone M5/114.15.2; PE-Ly6C, clone HK1.4; FITC-Ly6G, clone 1A8; FITC-Gr-1, clone RB6-8C5; PE-Cy7- or BV510-CD45.2, clone 104; PE-Cy7-CD80, clone 16-10A1; V510-CD86 clone GL-1; PE-CD163, clone S15049I; FITC-CD206, clone C068C2; APC-IFNg, clone XMG1.2; FITC- TNFa, clone MP6-XT22 were purchased from Biolegend. The purified F4/80 was diluted by 1:200 for IHC. mouse antibody Alex488-iNOS, clone CXNFT was purchased from ebioscience.<br>2. human antibody: PE-CD14, clone 63D3; BV510-CD80, clone 2D10; PerCP-Cy5.5-CD86, clone BU63, APC-CD163, clone GHI/61, FITC-CD206, clone 15-2 were purchased from Biolegend. FITC-iNOS, clone 4E5 was from Novus Biologicals.<br>3. Antibody PE-ARG1 for both human and mouse, clone A1exF5 was purchased from ebioscience.<br>4. TA99 antibody was made in our lab based on our previous studies.<br>5. WB antibodies: pSRC (#6943)and HRP-beta-tubulin (#5346) were from Cell Signaling Technology; pSIK (#ab199474) was from ABcam.<br>The dilution of all Conjugated antibodies from Biolegend, ebioscience and Novus Biologicals were indicated in the Methods and are freely available from manufacturer's websites.
Validation	TA99 in vivo dosage was validated based on our previous publication (Ref. 33).<br>All commercial antibodies were validated by the manufacture and commonly used in our lab. Information about host species, reactivity, and applications are freely available from manufacturer's websites.

PerCP-Cy5.5-CD11b, Rat antibody for flow cytometry in mouse and human. Validation and quality control test by immunofluorescent staining with flow cytometric analysis according to the manufacturer website. Product citations show as: https://www.biolegend.com/en-us/products/percp-cyanine5-5-anti-mouse-human-cd11b-antibody-4257

APC-F4/80, Rat antibody for flow cytometry in mouse. Validation and quality control test by immunofluorescent staining with flow cytometric analysis according to the manufacturer website. Product citations show as: https://www.biolegend.com/en-us/products/apc-anti-mouse-f4-80-antibody-4071

APC-Cy7-MHC-II, Rat antibody for flow cytometry in mouse. Validation and quality control test by immunofluorescent staining with flow cytometric analysis according to the manufacturer website. Product citations show as: https://www.biolegend.com/en-us/products/apc-cyanine7-anti-mouse-i-a-i-e-antibody-5966

PE-Ly6C, Rat antibody for flow cytometry in mouse. Validation and quality control test by immunofluorescent staining with flow cytometric analysis according to the manufacturer website. Product citations show as: https://www.biolegend.com/en-us/products/pe-anti-mouse-ly-6c-antibody-4904

FITC-Ly6G, Rat antibody for flow cytometry in mouse. Validation and quality control test by immunofluorescent staining with flow cytometric analysis according to the manufacturer website. Product citations show as: https://www.biolegend.com/en-us/products/fitc-anti-mouse-ly-6g-antibody-4775

FITC-Gr-1, Rat antibody for flow cytometry in mouse. Validation and quality control test by immunofluorescent staining with flow cytometric analysis according to the manufacturer website. Product citations show as: https://www.biolegend.com/en-us/products/fitc-anti-mouse-ly-6g-ly-6c-gr-1-antibody-458

PE-Cy7- or BV510-CD45.2, Mouse antibody for flow cytometry in mouse. Validation and quality control test by immunofluorescent staining with flow cytometric analysis according to the manufacturer website. Product citations show as: https://www.biolegend.com/en-us/products/brilliant-violet-510-anti-mouse-cd45-2-antibody-7998

PE-Cy7-CD80, American Hamster antibody for flow cytometry in mouse and dog cross-reactivity. Validation and quality control test by immunofluorescent staining with flow cytometric analysis according to the manufacturer website. Product citations show as: https://www.biolegend.com/en-us/products/pe-anti-mouse-cd80-antibody-43

BV510-CD86, Rat antibody for flow cytometry in mouse. Validation and quality control test by immunofluorescent staining with flow cytometric analysis according to the manufacturer website. Product citations show as: https://www.biolegend.com/en-us/products/brilliant-violet-510-anti-mouse-cd86-antibody-8745

PE-CD163, clone S15049I; Rat antibody for flow cytometry in mouse. Validation and quality control test by immunofluorescent staining with flow cytometric analysis according to the manufacturer website. Product citations show as: https://www.biolegend.com/en-us/products/apc-anti-human-cd163-antibody-6276

FITC-CD206, Rat antibody for flow cytometry in mouse. Validation and quality control test by immunofluorescent staining with flow cytometric analysis according to the manufacturer website. Product citations show as: https://www.biolegend.com/en-us/products/fitc-anti-mouse-cd206-mmr-antibody-7318

APC-IFNg, Rat antibody for flow cytometry in mouse. Validation and quality control test by immunofluorescent staining with flow cytometric analysis according to the manufacturer website. Product citations show as: https://www.biolegend.com/en-us/products/apc-anti-mouse-ifn-gamma-antibody-993

FITC- TNFa, Rat antibody for flow cytometry in mouse. Validation and quality control test by immunofluorescent staining with flow cytometric analysis according to the manufacturerswebsite. Product citations show as: https://www.biolegend.com/en-us/products/fitc-anti-mouse-tnf-alpha-antibody-976

Alex488-iNOS, Rat antibody for human, mouse and rat. Validated for flow cytometry in mouse. Product citations show as: https://www.thermofisher.com/antibody/product/iNOS-Antibody-Monoclonal/53-5920-80

PE-CD14, Mouse antibody for flow cytometry in human. Validation and quality control test by immunofluorescent staining with flow cytometric analysis according to the manufacturerswebsite. Product citations show as: https://www.biolegend.com/en-us/products/pe-anti-human-cd14-antibody-12011

BV510-CD80, Mouse antibody for flow cytometry in human and Rhesus cross-reactivity. Validation and quality control test by immunofluorescent staining with flow cytometric analysis according to the manufacturer website. Product citations show as: https://www.biolegend.com/en-us/products/brilliant-violet-510-anti-human-cd80-antibody

PerCP-Cy5.5-CD86, Mouse antibody for flow cytometry in Human. Validation and quality control test by immunofluorescent staining with flow cytometric analysis according to the manufacturer website. Product information show as: https://www.biolegend.com/en-us/products/percp-cyanine5-5-anti-human-cd86-antibody-16102

APC-CD163, Mouse antibody for flow cytometry in Human, African Green, Cynomolgus, Rhesus. Validation and quality control

test by immunofluorescent staining with flow cytometric analysis according to the manufacturer website. Product citations show as: https://www.biolegend.com/en-us/products/apc-anti-human-cd163-antibody-6276

FITC-CD206, Mouse antibody for flow cytometry in Human, African Green, Cynomolgus, Rhesus. Validation and quality control test by immunofluorescent staining with flow cytometric analysis according to the manufacturer website. Product citations show as: https://www.biolegend.com/en-us/products/fitc-anti-human-cd206-mmr-antibody-2993

FITC-iNOS, the same clone of Alex488-iNOS, product details as in https://www.novusbio.com/products/inos-antibody-4e5_nbp2-22119f

PE-ARG1, Rat antibody for for flow cytometry in human and mouse, validated by flow cytometric analysis of normal human peripheral blood cells according to the manufacturer website.

#6943, Rabbit antibody for human and mouse, validate by WB according to the manufacturer website. Product citations show as: https://www.cellsignal.com/products/primary-antibodies/phospho-src-family-tyr416-d49g4-rabbit-mab/6943

#5346, Rabbit antibody for human and mouse, validate by WB according to the manufacturer website. Product citations show as: https://www.cellsignal.com/products/antibody-conjugates/b-tubulin-9f3-rabbit-mab-hrp-conjugate/5346

ab199474, Rabbit monoclonal to human SIK1 (phospho T182) + SIK2 (phospho T175) + SIK3 (phospho T163), validate in 393T cells by WB according to the manufacturer website.

Eukaryotic cell lines

Policy information about cell lines

Cell line source(s)

The melanoma cell line B16F10 was purchased from ATCC® (CRL-6475), human B-cell lymphoma cell line (GMB) was generated as described in our previous publication (Ref.17) and maintained in house.

Authentication

The cell lines were not authenticated.

Mycoplasma contamination

Cells were tested for mycoplasma contamination and were negative by PCR.

Commonly misidentified lines
(See ICLAC register)

B16F16 and GMB are not commonly misidentified cell lines.

Animals and other organisms

Policy information about studies involving animals; ARRIVE guidelines recommended for reporting animal research

Laboratory animals

Mouse colony C57BL/6J (stock #000664, male, 6-10 weeks) and NSG (stock #005557, male, 10-12 weeks) were purchased from Jackson Laboratory and housed in specific pathogen-free facilities at MIT under 12-h light dark cycles, controlled temperature (~23 °C) and 40~50% humidity with free access to food and water.

Wild animals

This study did not involve in wild animals.

Field-collected samples

This study did not involve in field-collected samples.

Ethics oversight

All animal studies and procedures are approved by the Massachusetts Institute of Technology’s Committee for Animal Care.

Note that full information on the approval of the study protocol must also be provided in the manuscript.

Human research participants

Policy information about studies involving human research participants

Population characteristics

Describe the covariate-relevant population characteristics of the human research participants (e.g. age, gender, genotypic information, past and current diagnosis and treatment categories). If you filled out the behavioural & social sciences study design questions and have nothing to add here, write "See above."

Recruitment

human fresh blood from blind healthy donors are from commercial sources (Research Blood Components, LLC)

Ethics oversight

Protocols are approved by IRB at MIT.

Note that full information on the approval of the study protocol must also be provided in the manuscript.

Flow Cytometry

Plots

Confirm that:

- [x] The axis labels state the marker and fluorochrome used (e.g. CD4-FITC).
- [x] The axis scales are clearly visible. Include numbers along axes only for bottom left plot of group (a 'group' is an analysis of identical markers).
- [x] All plots are contour plots with outliers or pseudocolor plots.
- [x] A numerical value for number of cells or percentage (with statistics) is provided.

Methodology

Sample preparation	Described in detail in Methods
Instrument	BD FACS Fortessa used for analysis, BD Aria III used for sorting
Software	BD FACSDiva software for collection and FloJo v8.8.7 for analysis
Cell population abundance	Tumor-associated macrophages were sorted directly from dissociated tumors based on F4/80+CD11B+Ly6G-CD45+. ~95% live cells were validated post sorting by F4/80+CD11b+. Human monocytes were purified from PBMC using the EasySep human monocyte enrichment kit (STEMCELL, #19059) with ~85% purity for in vitro macrophage differentiation.
Gating strategy	Gating strategy was indicated in the figure legends and supplementary figures

- [x] Tick this box to confirm that a figure exemplifying the gating strategy is provided in the Supplementary Information.
