# Supplementary material for: High-throughput phenotypic screen and transcriptional analysis identify new compounds and targets for macrophage reprogramming
Source: Nat Commun. 2021 Feb 3;12:773. doi: 10.1038/s41467-021-21066-x (PMC7858590; doi:10.1038/s41467-021-21066-x)
Supplement: Supplementary file 4 — Description of Additional Supplementary Files [file 41467_2021_21066_MOESM4_ESM.pdf]

Description of Additional Supplementary Files

File Name: SupplementaryData1.xlsx
Description: Information on compounds used in the phenotypic screen and image quantification data

File Name: SupplementaryData2.xlsx
Description: Pathway analysis of proteins targeted by identified compounds

File Name: SupplementaryData3.xlsx
Description: The differentially expressed genes of hMDM response to compunds for Figure 2d.

File Name: SupplementaryData4.xlsx
Description: The differentially expressed genes of hMDM response to ligands for Figure 2f.

File Name: SupplementaryData5.xlsx
Description: The differentially expressed genes of hMDM response to compounds for Figure 4.

File Name: SupplementaryData6.xlsx
Description: GO enrichment network of top 10% central hub genes associated with macrophage activation.

File Name: SupplementaryData7.xlsx
Description: The differentially expressed genes of tumor macrophage induced by thiostrepton *in vivo*.
